# Pathological subtypes and sampling strategies determine diagnostic sensitivity in cervical lymph node tuberculosis: a retrospective study

**DOI:** 10.3389/fcimb.2025.1662518

**Published:** 2025-09-09

**Authors:** Xiaoyu Liu, Xuan Wang, Yuejie Li, Qibin Liu, Chao Quan, Xiyong Dai

**Affiliations:** Wuhan Pulmonary Hospital, Wuhan Institute for Tuberculosis Control, Wuhan, Hubei, China

**Keywords:** cervical lymph node tuberculosis, tuberculosis, etiological diagnosis, sampling, GeneXpert

## Abstract

**Objective:**

To investigate how pathological types and sampling methods affect positivity rates of five diagnostic techniques in cervical lymph node tuberculosis.

**Methods:**

We retrospectively analyzed 198 surgically confirmed cervical lymph node tuberculosis patients from Wuhan Pulmonary Hospital. Cases were stratified by pathological subtypes and collection methods. The specimens were tested using acid-fast bacillus smear microscopy, mycobacterium tuberculosis culture, quantitative polymerase chain reaction for tuberculosis DNA, simultaneous amplification and testing for tuberculosis, or GeneXpert.

**Results:**

All 198 cases showed granulomatous inflammation. Liquefactive necrosis occurred in 91.92% (182/198) of cases, with caseous necrosis in 87.88% (174/198), adjacent soft-tissue necrosis in 57.07% (113/198), and suppurative inflammation in 20.20% (40/198). Solid alterations without liquefactive necrosis (coagulative necrosis/non-necrotizing lymphadenitis) comprised 8.08% (16/198). The overall etiological positivity rate was 90.40% (179/198). GeneXpert showed highest sensitivity (90.36%), followed by tuberculosis DNA (74.24%), simultaneous amplification and testing (40.22%), Mycobacterium tuberculosis culture (16.67%), and acid-fast bacillus smear (14.72%). Among 33 culture-positive cases, 32 (96.97%) were GeneXpert positive. Rifampicin resistance detected by GeneXpert was 5.62% (10/178). In specimens with caseous necrosis, soft-tissue necrosis, or liquefactive necrosis, GeneXpert positivity significantly exceeded tuberculosis DNA (all P < 0.01). Liquefactive necrosis samples showed higher positivity than solid-change specimens for all techniques except culture (all P < 0.001). Drainage specimens yielded higher tuberculosis DNA and GeneXpert positivity than surgical resection specimens. Combining surgical and drainage specimens increased culture positivity to 26.09%.

**Conclusion:**

Etiological positivity rates in cervical lymph node tuberculosis correlate with pathological features. Maximizing liquefactive necrosis sampling for the GeneXpert assay and combining different sampling techniques (such as, surgical resection, incision and drainage, needle biopsy) for etiological detection enhances diagnostic accuracy.

## 1 Introduction

The 2024 global tuberculosis report identifies China as a high-burden country for tuberculosis (TB) and multidrug-resistant TB (MDR-TB), accounting for 7.3% of global rifampicin-resistant TB (RR-TB) cases (World Health Organization, 2024). Cervical (neck) tuberculous lymphadenitis constitutes 30-40% of extrapulmonary tuberculosis (EPTB) cases (Ganchua et al., 2020; Li et al., 2023; Zhao et al., 2024) and is characterized by localized chronic infection, granulomatous inflammation, and caseous necrosis (Flyger et al., 2020). Despite its clinical prevalence, diagnostic challenges persist, particularly in general hospitals where the proportion of extrapulmonary tuberculosis diagnoses remains relatively low. In contrast, specialized tuberculosis centers report higher diagnostic rates due to enhanced resources and professional capabilities (Ohene et al., 2019; Fang et al., 2022). This disparity arises from insufficient clinical vigilance, limited access to molecular diagnostics, and the histopathological mimicry of granulomatous lesions by non-tuberculous mycobacterial infections, sarcoidosis, and fungal diseases (Ohene et al., 2019; Yenilmez et al., 2021; Jain et al., 2024). Consequently, the absence of microbiological confirmation in certain patients undergoing surgical resection of cervical lymph nodes results in delayed diagnosis and suboptimal management of drug-resistant tuberculosis (DR-TB) (Lekhbal et al., 2020; Soriano et al., 2024). Improving etiological detection rates and the early identification of drug resistance remains a critical challenge (Ben Ayed et al., 2019; Li et al., 2023). This study investigates how pathological types and sampling methods influence etiological positivity rates in cervical tuberculous lymphadenitis (CTL) and proposes strategies to enhance diagnostic accuracy.

## 2 Methods

### 2.1 Study population

This retrospective single-center observational study, approved by the Ethics Committee of Wuhan Pulmonary Hospital (No. 2022057), included 198 patients, consisting of 71 males and 127 females, with a median age of 30 years. The basic information of the cases included in this study is shown in **Table 1**. All patients were clinically diagnosed with cervical lymph node tuberculosis, and surgical histopathology confirmed granulomatous inflammation between January 2023 and January 2024 in our surgical department. The diagnostic criteria for cervical tuberculous lymphadenitis were based on two pathways: a definitive diagnosis requires etiological confirmation through the detection of mycobacterium tuberculosis (MTB) in pathological specimens via any of five microbiological assays—acid-fast bacilli (AFB) staining, Löwenstein-Jensen culture for detection of MTB (MTB-culture), TB DNA detection by real-time quantitative polymerase chain reaction (PCR), GeneXpert MTB/RIF assay (Xpert), or simultaneous amplification and testing for tuberculosis (SAT-TB; RNA isothermal amplification with real-time fluorescence detection). The five detection techniques were implemented according to the methodology described in the studies by Liu and Wei et al (Liu et al., 2021; Wei et al., 2025). The criteria for each histopathological subgroup are defined as follows: (1) caseous necrosis: characterized by complete necrosis, microscopically presenting as homogeneous, dense, amorphous, eosinophilic areas, no discernible cellular outlines or ghosts visible; (2) necrosis involving adjacent soft tissue: microscopic evidence of necrotic components within the connective tissues (such as perinodal adipose tissue or fibrous tissue) surrounding the lymph node; (3) abscess/suppurative inflammation: lesion areas predominantly infiltrated by neutrophils; (4) coagulative necrosis: microscopically appearing as homogeneous, eosinophilic areas; however, unlike caseous necrosis, outlines or ghosts of necrotic cells remain discernible; (5) non-necrotizing lymphadenitis: microscopic examination reveals the formation of granulomatous nodules within the affected lymph node(s), but no areas of necrosis are identified.

**Table 1:** Clinical and demographic characteristics of 198 tuberculosis patients.

Clinical characteristics	Total (N = 198)
Sex	
Female	127 (64.14%)
Male	71 (35.86%)
Age, median (IQR), years	30 (24, 42)
Duration of illness (months)	2 (1.5, 2.5)
PPD skin test	
Strongly positive	85
Moderately positive	21
Negative	2
Preoperative anti-TB treatment duration	
1 month	117
1 month - 2 months	42
2 months	39
Presence of pulmonary TB	
Yes	84
No	114
ESR(mm/h)	17.0 (9.6, 30.0)
hCRP(mg/L)	2.605 (1.203,8.128)
198 cervical LNTB	198
PTB	84
other EPTB	29
cervical LNTB (isolated)	102
PTB and other EPTB overlap	17
Positive pulmonary TB Pathology (Sputum)	14.3 (12/84)

PPD, Purified Protein Derivative (tuberculin skin test). ESR, Erythrocyte Sedimentation Rate. hCRP, High-sensitivity C-reactive Protein. TB, Tuberculosis. IQR, Interquartile Range. LNTB, lymph node tuberculosis. PTB, Pulmonary Tuberculosis. EPTB, Extrapulmonary Tuberculosis. Other EPTB means excluding cervical LNTB. PPD test results interpretation criteria: ① Negative: Induration with an average diameter <5 mm or no reaction. ② Moderately positive: Induration with an average diameter ≥10 mm and <15 mm. ③ Strongly positive: Induration with an average diameter ≥15 mm, or local signs such as a double ring, blisters, necrosis, or lymphangitis.

A specimen is deemed positive if any single method yields a positive result. Clinical diagnosis is established through histopathological evidence of granulomatous inflammation, combined with at least one of the following: (1) concurrent pulmonary or extrapulmonary tuberculosis at other sites, (2) a strongly positive tuberculin skin test (PPD ≥15 mm, or the presence of double rings, blisters, necrosis, and lymphangitis at the local site) or T-SPOT positive, or (3) radiological or clinical evidence of lesion reduction following preoperative anti-tuberculosis therapy. Patients who tested positive using Gene Xpert MTB/RIF but negative in MTB culture were monitored until a definitive diagnosis was established. All surgically excised or biopsied specimens were examined by AFB smear microscopy, TB-DNA, SAT-TB, MTB-culture and X-pert MTB/RIF rapid molecular detection.

### 2.2 Statistical analysis

Data were analyzed using SPSS version 25.0. Chi-square and McNemar’s tests were employed to compare positivity rates, while Kappa statistics were utilized to evaluate concordance (K ≤ 0.4: poor; 0.4 < K ≤ 0.75: moderate; K > 0.75: strong). A p-value of < 0.05 was considered indicative of a statistically significant difference.

## 3 Results

### 3.1 Clinical characteristics and etiological findings in patients with cervical (neck) tuberculous lymphadenitis

A total of 198 patients (71 males and 127 females) with a median age of 30 years were included in this study. All 198 cases were diagnosed with cervical tuberculous lymphadenitis. Among these, 84 cases had coexisting pulmonary tuberculosis (PTB), while 29 cases had other forms of EPTB, excluding cervical lymph node tuberculosis. This included 17 cases that overlapped with both PTB and other EPTB. Isolated cervical lymph node tuberculosis occurred in 102 cases. The basic characteristics of the study population are presented in **Table 1**.

The overall positivity rate for the five detection methods applied to surgical specimens was 90.40% (179/198). Significant variations were observed across the detection methods: Xpert exhibited the highest positivity rate at 90.36% (178/197), followed by TB-DNA at 74.24% (147/198), SAT-TB at 40.22% (72/179), MTB culture at 16.67% (33/198), and AFB at 14.72% (29/197). Among the 147 TB-DNA-positive cases, 100% were also positive by Xpert. Of the 33 MTB culture-positive cases, 32 (96.97%) tested positive via the Xpert assay (see **Table 2**). Using MTB culture-positive cases as a reference, SAT-TB demonstrated a negative predictive value of 91.59% (98/107) and a positive predictive value of 25.00% (18/72).

**Table 2:** Positive detection rates of five diagnostic methods in patients with cervical tuberculous lymphadenitis.

Detection methods	Total (N = 198)
AFB	14.72% (29/197)
TB-DNA	74.24% (147/198)
SAT-TB	40.22% (72/179)
X-pert	90.36% (178/197)
MTB culture	16.67% (33/198)
Positive for any of the above five detection methods	90.4% (179/198)

For details of each detection method, refer to the Methods section in the main text. AFB, Acid-Fast Bacilli (staining). TB-DNA, Mycobacterium tuberculosis DNA. SAT-TB, Simultaneous Amplification and Testing for Tuberculosis. Xpert, GeneXpert MTB/RIF assay. MTB culture, Mycobacterium tuberculosis culture (Löwenstein-Jensen medium).

### 3.2 Drug resistance detection in cervical tuberculous lymphadenitis using Xpert MTB/RIF detection and MTB culture

In this study, 179 patients underwent simultaneous testing with five pathogen detection methods (AFB, TB-DNA, SAT-TB, Xpert, and MTB culture). Xpert detected rifampicin resistance in 5.62% (10/178) of the Xpert-positive cases, with 1 (10.00%) of these also being positive in the MTB culture assay. The MTB culture assay identified drug resistance in 21.21% (7/33) of cases, including resistance to isoniazid (5 cases), streptomycin (3 cases), ethambutol (2 cases), pyrazinamide (2 cases), rifampicin (1 case), and fluoroquinolones (1 case).

### 3.3 Pathological characteristics of cervical tuberculous lymphadenitis

**Figure 1:**
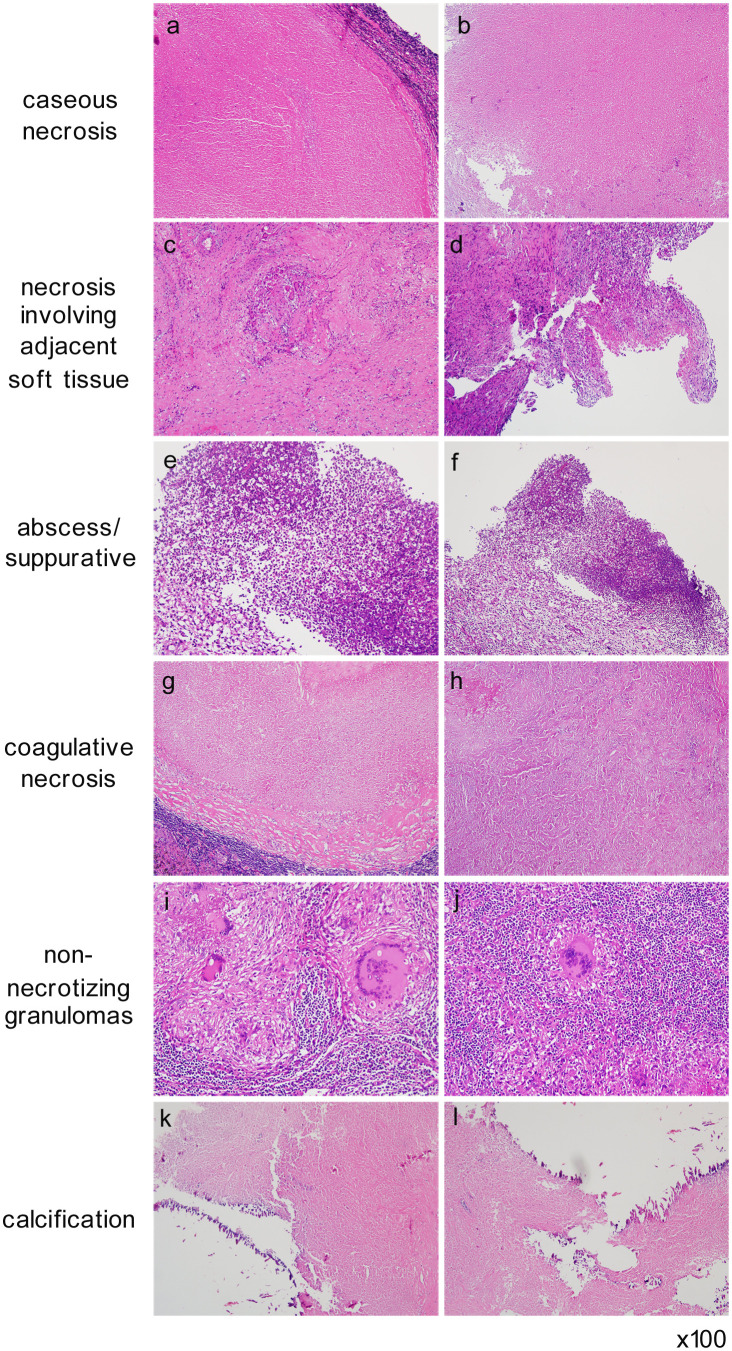
Typical pathological features in patients with cervical tuberculous lymphadenitis. Representative histopathological subtypes in the patient with cervical tuberculous lymphadenitis demonstrating granulomatous inflammation: caseous necrosis **(a, b)**, necrosis with adjacent soft tissue involvement **(c, d)**, abscess/suppurative inflammation **(e, f)**, coagulative necrosis **(g, h)**, non-necrotizing lymphadenitis **(i, j)**, and calcification **(k, l)**.

The cohort of 198 patients was stratified into five subgroups based on distinct histopathological findings: caseous necrosis, necrosis with adjacent soft tissue involvement, abscess/suppurative inflammation, coagulative necrosis, and non-necrotizing lymphadenitis. The analysis of pathological subgroups revealed the following findings: caseous necrosis was observed in 87.88% (174/198) of cases, necrosis involving adjacent soft tissue in 57.07% (113/198), abscess/suppurative inflammation in 20.20% (40/198), coagulative necrosis in 9.60% (19/198), and non-necrotizing lymphadenitis in 1.52% (3/198). Liquefactive necrosis, which includes caseous necrosis, abscess/suppurative inflammation, or necrosis involving adjacent soft tissue, was present in 91.92% (182/198) of cases. In contrast, pathological solid alterations, encompassing coagulative necrosis and non-necrotizing lymphadenitis, accounted for 11.11% (22/198) of the cases; among these, 6 cases demonstrated coexisting liquefactive necrosis, while 16 cases exhibited solid alterations without liquefactive necrosis. Additionally, calcification was observed in 7.07% (14/198) of the specimens, with all calcified cases coexisting with caseous necrosis (**Table 3**). Representative pathological features are illustrated in **Figure 1** (Hematoxylin and Eosin staining).

**Table 3:** Pathological Findings in patients with cervical tuberculous lymphadenitis.

Pathological feature	Total (n=198)
Liquefactive necrosis	91.92% (182/198)
Caseous necrosis	87.88% (174/198)
Necrosis involving adjacent soft tissue	57.07% (113/198)
Abscess/suppurative inflammation	20.20% (40/198)
Solid alterations without liquefactive necrosis	8.08% (16/198)
Coagulative necrosis	6.57% (13/198)
Non-necrotizing lymphadenitis	1.52% (3/198)
Solid alterations with liquefactive necrosis	3.03% (6/198)
Solid alterations (with or without liquefactive necrosis)	11.11% (22/198)
Coagulative necrosis	9.60% (19/198)
Non-necrotizing lymphadenitis	1.52% (3/198)
Calcification	7.07% (14/198)

Due to overlapping pathological subtypes among patients, the sum exceeds 198.

### 3.4 Association between different pathological features and positivity rates of etiological detection methods in cervical tuberculous lymphadenitis

The cohort of 179 patients, all subjected to concurrent testing with five pathogen detection modalities, was classified into five subgroups according to distinct histopathological categories. Within each pathological subgroup, Xpert exhibited the highest positivity rate (89.94%) among the five pathogen detection methods, followed by TB-DNA (75.41%). The detection rate of Xpert was significantly higher than that of TB-DNA across all five pathological subtypes (all *P* < 0.01). Regardless of liquefactive necrosis subtypes—caseous necrosis, necrosis involving soft tissue, or abscess/suppurative inflammation—Xpert maintained MTB positivity rates exceeding 90% across all subcategories. Furthermore, the Xpert assay demonstrated a markedly high MTB positivity rate of 95.09% in liquefactive necrosis specimens, compared to 37.5% in those with solid alterations (*P* < 0.001). Furthermore, pathological specimens exhibiting liquefactive necrosis showed significantly higher detection rates in AFB staining, TB-DNA, and SAT-TB assays compared to specimens with solid-caseous changes alone (all *P* < 0.001) (**Table 4**).

**Table 4:** Impact of different pathological subtypes of cervical tuberculous lymphangitis on positivity rates of different diagnostic assays.

Pathological subtype	AFB	TB-DNA	SAT-TB	X-pert	MTB-culture	X-pert vs TB-DNA (P value)
Caseous necrosis (157)	15.29% (24/157)	81.53% (128/157)	41.40% (65/157)	96.18% (151/157)	17.20% (27/157)	<0.001
Necrosis involving adjacent soft tissue (101)	16.83% (17/101)	83.17% (84/101)	49.50% (50/101)	96.04% (97/101)	16.83% (17/101)	0.006
Abscess/suppurative inflammation (37)	21.62% (8/37)	86.49% (32/37)	45.95% (17/37)	91.89% (34/37)	13.51% (5/37)	0.079
Liquefactive necrosis (163)	15.95% (26/163)	80.98% (132/163)	42.33% (69/163)	95.09% (155/163)	16.56% (27/163)	0.004
Solid alterations (16)	0 (0/16)	18.75% (3/16)	12.50% (2/16)	37.50% (6/16)	6.25% (1/16)	0.235
Liquefactive necrosis vs solid alterations (P value)	<0.001	<0.001	<0.001	<0.001	0.47	

AFB, Acid-Fast Bacilli. TB-DNA, Mycobacterium tuberculosis DNA (quantitative PCR). SAT-TB, Simultaneous Amplification and Testing for Tuberculosis. Xpert, GeneXpert MTB/RIF assay. MTB culture, Mycobacterium tuberculosis culture (Löwenstein-Jensen medium).

Among 163 specimens with liquefactive necrosis, TB-DNA positivity was observed in 80.98% (132/163). Notably, 157 specimens exhibited concomitant caseous necrosis (TB-DNA positivity: 81.53%, 128/157), with none showing coexisting coagulative necrosis. In contrast, the remaining 6 specimens without caseous necrosis demonstrated lower TB-DNA positivity (66.67%, 4/6), accompanied by adjacent soft tissue involvement (4 cases), abscess formation (3 cases), and concurrent coagulative necrosis (5 cases).

### 3.5 Impact of sampling methods on etiological positivity rates in cervical tuberculous lymphadenitis

Among 27 patients diagnosed with cervical tuberculous lymphadenitis who underwent both surgical resection and needle biopsy, a comparative analysis of paired surgical and biopsy specimens utilizing identical microbiological assays revealed that the Xpert method exhibited a significantly higher MTB positivity rate in surgical specimens (*P* = 0.005). However, no statistically significant differences were observed between surgical resection and biopsy specimens for AFB, TB-DNA, or MTB-culture methods, with SAT-TB excluded due to insufficient sample size (**Table 5**). Additionally, a comparative analysis of 27 paired surgical resection specimens and incision/drainage specimens using the same diagnostic protocols demonstrated that TB-DNA and MTB culture methods yielded higher positivity rates in incision/drainage specimens compared to surgical resection specimens (both *P* = 0.002). Specifically, there was no difference in the positivity rate of pathogens detected by AFB staining and Xpert testing between paired surgical resection specimens and incision/drainage specimens (**Table 6**).

**Table 5:** Impact of sampling methods on etiological positivity rates in patients with cervical tuberculous lymphadenitis.

Test method	AFB (n=20)		TB-DNA (n=23)		Xpert (n=26)		MTB culture (n=24)	
Sample collection method	Puncture biopsy- positive	Puncture biopsy- negative	Puncture biopsy- positive	Puncture biopsy- negative	Puncture biopsy- positive	Puncture biopsy- negative	Puncture biopsy- positive	Puncture biopsy- negative
Surgical resection-positive	1	3	17	1	23	2	1	2
Surgical resection-negative	3	13	5	0	0	1	2	19
P value^a^	1		0.219		0.5		1	
Kappa Value	0.063		0.078		0.469		0.238	
P value	0.78		0.59		0.005		0.243	

AFB, Acid-Fast Bacilli. TB-DNA, Mycobacterium tuberculosis DNA. Xpert, GeneXpert MTB/RIF assay. MTB culture, Mycobacterium tuberculosis culture (Löwenstein-Jensen medium). P value^a^ represent MC test. P value indicates the statistical difference between the two sample collection methods based on the chi-square test.

**Table 6:** Impact of sampling methods on etiological positivity rates in patients with cervical tuberculous lymphadenitis.

Test method	AFB (n=18)		TB-DNA (n=27)		Xpert (n=26)		MTB culture (n=23)	
Sample collection method	I&D positive	I&D negative	I&D positive	I&D negative	I&D positive	I&D negative	I&D positive	I&D negative
Surgical resection-positive	1	1	22	0	26	0	3	0
Surgical resection-negative	3	13	3	2	0	0	3	17
P value^a^	0.625		0.25				0.25	
Kappa Value	0.217		0.521		1		0.596	
P value	0.316		0.002				0.002	

AFB, Acid-Fast Bacilli. TB-DNA, Mycobacterium tuberculosis DNA. Xpert, GeneXpert MTB/RIF assay. MTB culture, Mycobacterium tuberculosis culture (Löwenstein-Jensen medium). I&D, Incision and Drainage. P value^a^ represent MC test. P value indicates the statistical difference between the two sample collection methods based on the chi-square test.

Combining needle biopsy specimens with surgical resection specimens resulted in increased positivity rates for AFB (35.00%, 7/20), TB-DNA (100%, 23/23), Xpert (96.15%, 25/26), and MTB culture (20.83%, 5/24) (**Table 5**). Similarly, pairing incision/drainage specimens with surgical specimens elevated positivity rates for AFB (27.78%, 5/18), TB-DNA (92.59%, 25/27), Xpert (100%, 26/26), and MTB culture (26.09%, 6/23) (**Table 6**).

## 4 Discussion

Granulomatous inflammation represents a non-specific pathological feature observed not only in tuberculous lymphadenitis but also in non-tuberculous mycobacterial infections, sarcoidosis, cat-scratch disease, and certain fungal infections (Öztomurcuk et al., 2022). Consequently, the presence of granulomatous inflammation alone cannot establish a definitive diagnosis of tuberculous lymphadenitis. The pathological progression of cervical tuberculous lymphadenitis demonstrates significant complexity: initial caseous necrosis may evolve into liquefactive abscess formation (Srinivas and Nair, 2019), and capsular rupture of necrotic components can lead to soft tissue infiltration, while calcification typically occurs during the healing phase (Yang and Du, 2019). Nevertheless, atypical manifestations such as coagulative necrosis or non-necrotizing lymphadenitis may emerge in scenarios involving robust host immunity, early disease stages, or low bacterial burden (Srinivas and Nair, 2019; Yang and Du, 2019). The diagnosis of cervical tuberculous lymphadenitis presents multiple challenges: firstly, the non-specificity of pathological characteristics (Shah et al., 2017); secondly, the persistent inability to achieve etiological confirmation even in partially resected lymph node specimens (Kim et al., 2025). These limitations not only compromise treatment adherence but may also delay the diagnosis and management of drug-resistant tuberculosis (Wulandari et al., 2024). Therefore, enhancing diagnostic yield in extrapulmonary tuberculosis specimens remains imperative. Through systematic analysis of 198 surgical cases with cervical tuberculous lymphadenitis, this study pioneers the identification of pathological subtypes and sampling methodologies as critical determinants influencing etiological detection outcomes, thereby proposing novel strategies to address diagnostic dilemmas in extrapulmonary tuberculosis.

Current clinical diagnostic techniques for TB exhibit varying sensitivities and clinical utilities. AFB staining demonstrates low positivity rates in specimens obtained from cervical tuberculous lymphadenitis. As highlighted in the study by Mukhida et al., extrapulmonary TB specimens such as pleural fluid show Ziehl-Neelsen staining positivity rates of merely 0-40%, which aligns with our findings (Mukhida et al., 2025). Notably, when AFB-positive results coincide with TB-DNA negativity, this combination may indicate potential non-tuberculous mycobacterial infections (Kunkel et al., 2016). Published evidence reveals that SAT-TB achieves higher detection rates than AFB in morning sputum specimens from pulmonary TB cases, with positive results suggesting mycobacterial viability and demonstrating strong concordance with clinically confirmed TB diagnoses (Yan et al., 2018). Our findings indicate that SAT-TB exhibits lower sensitivity compared to the Xpert assay, which precludes its use as a first-line diagnostic tool for cervical tuberculous lymphadenitis. However, its high negative predictive value (91.59%) suggests potential utility as a predictive biomarker for MTB culture-negative status in lymph node specimens. Meanwhile, TB-DNA targeting species-specific nucleic acid sequences, exhibits superior specificity alongside favorable cost-effectiveness and accessibility in routine clinical practice (Kim et al., 2023). The Xpert MTB/RIF assay demonstrates high sensitivity and specificity, while also providing evidence for rifampicin resistance. It has been widely utilized in diagnosing pulmonary tuberculosis and osteoarticular tuberculosis (Sun et al., 2019). In our study, the GeneXpert MTB/RIF assay achieved an etiological positivity rate of 90.36% (*P* < 0.001 vs. other methods). The elevated positive detection rate for etiology is likely attributable to our “liquefactive necrosis-priority” sampling principle. Previous studies have demonstrated that the Xpert MTB/RIF assay exhibits superior diagnostic capability for rifampicin resistance detection compared to the MTB-culture assay (Mukhida et al., 2022). Our study revealed consistent results between Xpert rifampicin resistance determinations and MTB-culture drug susceptibility testing. Therefore, the Xpert assay is recommended as the preferred method for both diagnosing cervical lymph node tuberculosis and initial screening for rifampicin resistance. Although the positive detection rate of MTB culture was only 16.67%, but the drug susceptibility testing results obtained from it play a pivotal role in guiding the formulation of subsequent treatment regimens.

Among 179 specimens subjected to five etiological detection methods, those exhibiting liquefactive necrosis demonstrated significantly higher etiological detection rates via AFB, TB-DNA, SAT-TB, and Xpert methods compared to specimens with only solid alterations. This indicates a strong correlation between microbiological positivity rates and pathological alterations. In abscess-type pathological subtypes, AFB achieved a positivity rate exceeding 20%, while TB-DNA surpassed 85%. Moreover, Xpert exhibited a 100% detection rate in incision/drainage specimens from abscesses. This enhanced sensitivity likely arises from the abundant necrotic material characteristic of abscess formation. Furthermore, specimens with coexisting coagulative necrosis and liquefactive necrosis showed a lower TB-DNA positivity rate (66.67%) compared to pure liquefactive necrosis specimens (81.53%) (*P* = 0.032). Collectively, these findings suggest that a greater proportion of liquefactive necrosis correlates with higher microbiological yield and MTB burden. These results align with reports indicating enhanced MTB survival in necrotic neutrophils compared to intact cells (Liang et al., 2020). These findings also indicates that neutrophil hydrolases break down necrotic matrices, releasing MTB antigens or DNA, thereby enhancing the sensitivity of molecular tests such as PCR. Additionally, MTB inhibits neutrophil apoptosis, prolonging neutrophil survival and further promoting bacterial proliferation (Andrews et al., 2024). Compared to other fluid necrosis specimens, calcified liquid necrosis specimens exhibit lower pathogen detection rates across various testing methods (**Supplementary table 1**). This may be related to the calcified encapsulation of necrotic material, which prevents pathogens from being fully released by conventional digestive solutions (Sharma et al., 2022). The underlying mechanisms and this conclusion require further in-depth investigation.

Therefore, we recommend prioritizing specimens exhibiting liquefactive necrosis from cervical lymphadenitis for laboratory testing. To optimize the acquisition of liquefactive necrotic specimens, the following recommendations are proposed. Firstly, clinicians should perform color contrast-enhanced ultrasound punctures to accurately access liquefied necrotic zones, guided by real-time hypoechoic imaging findings (Zhang et al., 2023). The Xpert system demonstrated a diagnostic yield of 93.3% (237/254) for etiological identification in specimens obtained via contrast-enhanced ultrasound-guided biopsy (Sun et al., 2021). Secondly, surgical teams must prioritize the collection of caseous necrosis under direct visualization. Furthermore, for both fresh specimens and formalin-fixed paraffin-embedded tissue samples, pathologists should collaborate closely with laboratory technicians to maximize the selection of necrotic components. These findings provide theoretical support for optimizing specimen collection strategies and enhancing pathogen detection rates.

Our findings indicate that specimens obtained through incision and drainage exhibit higher rates of TB-DNA positivity and Xpert assay detection compared to surgical specimens. These differences may be attributed to the enhanced accessibility of deep necrotic tissue during incision and drainage procedures. Furthermore, the combination of specimens from incision and drainage with surgical resection specimens, or the integration of puncture specimens with surgical resection specimens, results in higher overall pathogen detection rates than employing a single sampling method. This is particularly critical for MTB-culture diagnostic approaches, which inherently have low etiological positivity rates. Therefore, clinicians are advised to adopt a multimodal sampling strategy (e.g., combining incision and drainage with surgical resection sampling) for MTB-culture testing to enhance detection sensitivity for pathogen.

### 4.1 Study limitations and future directions

This study has some limitations. Firstly, it employs a single-center design, which may limit the generalizability of the findings. Secondly, the intraoperative visual assessment of small necrotic foci, such as micro-abscesses, conducted by surgeons may introduce subjective bias. Thirdly, the relationship between varying proportions of necrosis and drug resistance was not evaluated. In future research, we plan to increase the sample size and conduct multicenter validation of our conclusions. Additionally, we will further investigate the correlation between the area of necrosis and drug resistance. In summary, we aim for this study to serve as a foundation for improving the pathogen-positive rate of extrapulmonary tuberculosis.

## 5 Conclusion

This study highlights the critical role of pathological features and sampling techniques in optimizing the etiological detection of cervical lymph node tuberculosis. The Xpert assay demonstrated superior sensitivity (90.36%), significantly outperforming four other diagnostic techniques, particularly in specimens with liquefactive necrosis (caseous necrosis, abscess, or soft tissue involvement), where its positivity rate exceeded 95%. Pathological analysis revealed that specimens with liquefactive necrosis exhibited markedly higher detection rates across AFB, TB-DNA, SAT-TB, and Xpert assay compared to those with solid alterations, emphasizing the necessity of prioritizing necrotic tissue during sampling. Furthermore, combining incision/drainage specimens with surgical resection specimens improved MTB culture positivity rates, addressing its inherent low sensitivity. The Xpert assay also reliably detected rifampicin resistance, aligning with culture-based drug susceptibility results. These findings advocate for a stratified diagnostic approach, prioritizing the Xpert assay for specimens exhibiting liquefactive necrosis to maximize sensitivity. Additionally, employing a combination of multiple sampling techniques—such as surgical resection, incision and drainage, or needle biopsy—can enhance the positive rate of etiology. Such strategies are crucial in countries burdened by extrapulmonary tuberculosis (TB) as they expedite diagnosis, guide therapy, and mitigate the risks of drug resistance. Future multicenter studies should aim to validate these findings and explore the relationship between the extent of necrosis and resistance patterns to refine diagnostic frameworks.

## Data Availability

The raw data supporting the conclusions of this article will be made available by the authors, without undue reservation.

## Supplementary material

Supplementary Table 1 Pathogen positive rates in calcification specimens. AFB, Acid-Fast Bacilli. TB-DNA, Mycobacterium tuberculosis DNA (quantitative PCR). SAT-TB, Simultaneous Amplification and Testing for Tuberculosis. Xpert, GeneXpert MTB/RIF assay. MTB culture, Mycobacterium tuberculosis culture (Löwenstein-Jensen medium).
