# Supplementary material for: Pathological subtypes and sampling strategies determine diagnostic sensitivity in cervical lymph node tuberculosis: a retrospective study
Source: Front Cell Infect Microbiol. 2025 Sep 9;15:1662518. doi: 10.3389/fcimb.2025.1662518 (PMC12454339; doi:10.3389/fcimb.2025.1662518)
Supplement: Supplementary Table 1 — Pathogen positive rates in calcification specimens. AFB, Acid-Fast Bacilli. TB-DNA, Mycobacterium tuberculosis DNA (quantitative PCR). SAT-TB, Simultaneous Amplification and Testing for Tuberculosis. Xpert, GeneXpert MTB/RIF assay. MTB culture, Mycobacterium tuberculosis culture (Löwenstein-Jensen medium). [file DataSheet1.docx]

Supplementary Table 1

|  | AFB | TB-DNA | SAT-TB | X-pert | MTB-culture |
| --- | --- | --- | --- | --- | --- |
| Calcification | 7.14%  （1/14） | 78.57%  （11/14） | 7.69%  （1/13） | 85.71%  （12/14） | 7.14%  （1/14） |

Pathogen positive rates in calcification specimens. AFB, Acid-Fast Bacilli. TB-DNA, Mycobacterium tuberculosis DNA (quantitative PCR). SAT-TB, Simultaneous Amplification and Testing for Tuberculosis. Xpert, GeneXpert MTB/RIF assay. MTB culture, Mycobacterium tuberculosis culture (Löwenstein-Jensen medium).
